# Surgery

**Published:** 1899-10

**Authors:** John Parmenter, Nelson W. Wilson

**Affiliations:** Buffalo, N.Y.; Professor of anatomy and adjunct professor of clinical surgery, Buffalo University Medical College; Buffalo, N. Y.


### Progress in Medical Science.

### SURGERY.

Conducted by JOHN PARMENTER, M. D., Buffalo, N. Y.
Professor of anatomy and adjunct professor of clinical surgery, Buffalo University Medical College.
AND
NELSON W. WILSON, M. D., Buffalo, N. Y.

### AN INTERESTING BIT OF SURGERY.

ILLUSTRATING an interesting bit of surgical work, the *Lancet* recently reported that Dr. Abbott-Anderson treated a cook who, while sharpening a knife had cut off the tip of his nose. The patient came to the office without the fragment and a boy was sent to look for it. He returned with the piece which was "a thin strip an inch long and half an inch broad at its lower end." This piece was thoroughly washed in warm boric acid solution and stitched into place with fine black silk. It had been severed about half an hour. For four hours after the suturing the nose was kept covered with compresses wrung out in hot boric acid solution, and was then put up with a thick dressing. The wound healed by first intention.

---

### THE CAUSE OF DEATH BY BURNS.

At various times many theories regarding the cause of death by burns have been advanced. All have been more or less carefully investigated. Azzarello (*Giorn. Mal. delle Mal. Ven. e della Pelle*) reports the results of his very complete work in this direction. The theories worked on were the four most prominent: (1) Shock of extreme pain; (2) thrombosis, embolism, or destruction of red blood corpuscles; (3) pyemic infection from the burned surface; (4) poison formed by the action of heat on the tissues, or auto-intoxication from defective elimination by the skin.

Azzarello's investigations show the correctness of the intoxication theory. He eliminated shock by the administration of chloroform and by section of the nerves supplying the burned part. An animal which had been chloroformed died in the same time and with the same symptoms as one unanesthetised. Not the slightest difference was made by section of the nerves, and death came so rapidly that there was no time for bacterial infection. After death from burns, there was not found any destruction of red corpuscles, there was no embolism, nor any thrombosis. Following out the intoxication theory, Azzarello used the blood from animals which had been burned, and extracts of burned tissues, for injection into the healthy animals, which died with all the symptoms exhibited by those which had been burned. Injections of large quantities of artificial serum, seemed to save life in many instances.

---

### CELLULOID SUTURES AND LIGATURES.

PAGENSTECHER (*Deut. Med. Woch.*) takes a good thread, boils for half an hour in a 1 per cent. solution of soda, washes in boiling water, and dries between sterile compresses. It is then soaked in a solution of celluloid, and passed again through the same solution. Afterward it is sterilised by steam under pressure, and preserved for use either dry or in an alcoholic solution of bichloride of mercury. The threads have a smooth surface, never tangle, cannot absorb secretions, and are easily tied. Pagenstecher uses these celluloid threads to the exclusion of silk, and the use of catgut for ligatures has been greatly reduced. The results have been good and the saving considerable.

---

### A HORSEHAIR SCRUBBER.

FORBES (*Phil. Med. Jour.*) has gone to the printing office and applied to surgical use an article of the printer's toilet, which is so old that its origin is lost in the mazes of time. From time immemorial printers when they washed up have used what they called the "rat," which is nothing but a bunch of horsehair. This has cleansed countless of printers' ink and the grime of type metal. In fact, it is the only thing which will do the work.

Forbes uses a "rat" instead of a brush in cleansing the hands and preparing the operative field, and he has found a valuable use for it. The "rat" is stiff enough to scrape away dirt, but it will not injure the most delicate skin. One beauty of the "rat" is that it can be sterilised and is practically indestructible.

---

### NEW WAY OF CLOSING THE PERITONEUM.

O'Hara (*British Med. Jour.*) gives this method of treatment of peritoneal opening: The incision through the peritoneum is never made more than four or five inches in length. If additional room is required the opening is stretched. As the membrane is very elastic it readily lends itself to this procedure. When the operation is over, a purse-string suture of kangaroo tendon is passed completely around the opening in the peritoneum, beginning at the middle of the incision. When this is drawn up and tied the peritoneal cavity is perfectly closed against leakage from without, and there is no raw surface within to form adhesions to the intestinal coils. The suture is easily applied, and the peritoneum is in a far better condition to withstand any post-operative vomiting than after the ordinary suture. The remaining layers of the abdominal wall are closed by interrupted sutures. He has never seen any bad results follow this method of closure.

---

### EPILEPSY OPERATIONS A FAILURE.

Amat (*Bull. Gen. de Ther.*) gives a very interesting review of cases and records of resection of the cervical sympathetic for the cure of epilepsy, which shows the operation is most unsatisfactory. In many cases the cutting has been followed by an increase in the number of seizures. There was trophic disturbances in some patients a considerable time afterward. Amat advises against operation.

---

### AN INTERESTING SURGICAL CASE.

Balton (*Medical News*) reports an interesting surgical case. The patient was shot, the ball entering the abdomen, just to the right of the median line and about two and one-half inches below the tip of the ensiform cartilage. There was a considerable degree of shock, but no further symptoms significant of intestinal injury or injury of the lung. Temperature 100° F., respiration 26, pulse 106. On opening the peritoneal cavity the anterior surface of the left lobe of the liver presented a horizontal laceration two and one-half inches in length and one inch in depth, from which fairly active bleeding was going on. There was a considerable amount of free blood in the peritoneal cavity. Digital exploration of the liver wound failed to disclose the path of the bullet, and it was tightly packed with gauze, which easily checked the hemorrhage from it. On the sixth day the patient himself discovered the bullet beneath the skin overlying the sixth intercostal space in the right axillary line, and a small incision served to remove it from this position. The right pleural cavity contained some bloody fluid which was removed by aspiration and the patient made an excellent recovery.

There are two points of interest here: First, a wound of the lung without hemoptysis, and the apparent ease with which liver hemorrhage was arrested by packing with gauze. Balton suggests, inferentially, that gauze crowded into liver wounds and tight bandaging of the abdomen and lower ribs and enforcing costal breathing, to prevent dislodgment of the packing by diaphragmatic movements, is the proper procedure.

---

### ABSOLUTE REST FOR GUN-SHOT WOUNDS.

LUCAS-CHAMPIONNIERE, at the May meeting of the French Academy of Medicine, showed a man who two months previously had been shot in the chest. The only treatment accorded him was enforced rest and cleansing of the external wound. There was marked collapse and considerable expectoration of blood. Absolute rest is demanded in such cases. The patient should not be turned over for auscultation. The position should be horizontal; he may be slightly inclined if respiration is facilitated thereby. Morphine should be used for restlessness, laxatives as a general course, and if necessary stimulants should be resorted to.

---

### HYDATID CYSTS OF THE LIVER.

DIEULAFOY reports to the French Academy of Medicine two permanent cures of hydatid cysts of the liver. The first was by laparatomy and extirpation; the second, in a child, was by aspiration of the contents of the cyst.

Puncture is suitable only in recent cases. Great care should be exercised to prevent the escape of fluid into the peritoneal cavity. A fine needle should be used and the entire contents of the cyst drawn off at the first puncture. In the event of the needle clogging before all the fluid is withdrawn, introduce a second and then withdraw both at once.

Sieur reports to the Surgical Society of France that he resected the ununited ends of a clavicle and sutured with two threads of strong silk. The fracture was of eight months' standing and separated the outer third from the inner two-thirds. The arm was fixed for a month. In sixteen months all traces of fracture had disappeared and the arm's function was perfect.

THE TREATMENT OF HEMORRHOIDS.

An interesting discussion of the treatment of hemorrhoids occupied a recent meeting of French surgeons, and the sentiments of eminent men showed a wide difference of opinion. The *Medical News* gives the following condensation of the discussion :

Monod, operating under chloroform, does not touch the external hemorrhoids and cuts off the internal ones, one at a time, with scissors, suturing immediately the cut mucous edges of each. A drain, wrapped with iodoform gauze, is left in the anus.

Reclus uses cocaine and thinks it important to remove the external as well as the internal hemorrhoids.

Pozzi said that it is advisable to respect, as much as possible, the mucous membrane. He, therefore, employs only ignipuncture in the treatment of hemorrhoids.

Tillaux also uses the thermocautery in the same manner. He said that its only inconvenience is the pain which lasts sometimes for two or three days after its use.

Quenu advocated general anesthesia in order to keep the rectum of the patient still. Under cocain it is drawn up involuntarily, even though no pain be felt. He said that different operations were indicated in different cases. If hemorrhoids are particularly turgid, ignipuncture is a better treatment than excision. Prolapse of the mucous membrane is a common complication which cannot be cured by the cautery nor by partial excision. Ignipuncture gives eschars, which do not come away for twelve or fifteen days, and its one advantage is in the economy of blood. The sole objection to Whitehead's operation is the difficulty in carrying it out properly; but that will disappear if the surgeon will accustom himself to its details. It is not followed by pain, and cicatrisation is complete in two weeks. He had never seen any contractions follow this operation.

Routier said that there would be no danger of retraction if the line of junction of mucous membrane and skin was respected. His plan is to ligate each hemorrhoid at its base, and then remove the projecting portion with a thermocautery.

### SUTURE OF THE AXILLARY ARTERY.

RICARD, of Paris, in removing the axillary glands, accidentally incised the axillary artery. He applied a lateral suture of catgut at four places. The result was perfect, there being no difference in the radial pulse of the two arms.

This report brought from Tuffier the statement that in experimenting on animals, suture of an artery was usually followed by callous which so compressed the artery as to make it impervious. Brazy considered suturing of the axillary unnecessary as he had resected five inches of the artery with no ill-effects.

---

### INDICATIONS FOR OPERATION IN CHOLELITHIASIS.

NAUNYN (*Centralbl. f. Chir.*) affirms that cholelithiasis is not so grave as hospital practice leads one to think. A favorable issue results in the majority of cases without surgical interference. Stones as large as a cherry can pass the common duct, and in many cases a fistula forms between the common duct and the duodenum, and then still larger stones may pass. Indication for operation are given as follows :

1. It is not justifiable to advocate operation in every case as soon as a diagnosis of cholelithiasis has been established, for operative interference does not guarantee a permanent cure, since stones may be left behind, or others form.

2. Cases of acute cholecystitis with a greatly distended gall-bladder, as well as chronic cases of hydrops of the gall-bladder should be submitted to operation.

3. In cases of chronic recurrent cholelithiasis.

4. In cases of obstructive jaundice operation should not be resorted to until a thorough Carlsbad treatment has failed.

---

### A POINT IN INTESTINAL SUTURE.

FRAZER (*Lancet*) suggests a simple method to insure accurate union along the mesenteric line, in suture of the small intestine. He rotates the two ends of bowel for a slight distance in opposite directions, so that there may be room for two or three stitches between the cut ends of the mesentery. This insures that there shall be no place in the suture line where there shall not be at least one serous surface involved in the suture. The procedure can be carried out with any end-to-end anastomosis.

